# Leveraging change point detection to discover natural experiments in data

**DOI:** 10.1140/epjds/s13688-022-00361-7

**Published:** 2022-09-03

**Authors:** Yuzi He, Keith A. Burghardt, Kristina Lerman

**Affiliations:** 1 Information Sciences Institute, University of Southern California, Marina del Rey, CA USA; 2 Department of Physics and Astronomy, University of Southern California, Los Angeles, CA USA

**Keywords:** Change point detection, High-dimensional data, Regression discontinuity design, Causal effect

## Abstract

Change point detection has many practical applications, from anomaly detection in data to scene changes in robotics; however, finding changes in high dimensional data is an ongoing challenge. We describe a self-training model-agnostic framework to detect changes in arbitrarily complex data. The method consists of two steps. First, it labels data as before or after a candidate change point and trains a classifier to predict these labels. The accuracy of this classifier varies for different candidate change points. By modeling the accuracy change we can infer the true change point and fraction of data affected by the change (a proxy for detection confidence). We demonstrate how our framework can achieve low bias over a wide range of conditions and detect changes in high dimensional, noisy data more accurately than alternative methods. We use the framework to identify changes in real-world data and measure their effects using regression discontinuity designs, thereby uncovering potential natural experiments, such as the effect of pandemic lockdowns on air pollution and the effect of policy changes on performance and persistence in a learning platform. Our method opens new avenues for data-driven discovery due to its flexibility, accuracy and robustness in identifying changes in data.

## Introduction

The explosive growth of Big Data has transformed the study of human behavior [1]. Yet one critical use case, inferring the effect of policies and interventions, has proven challenging. To address this challenge, researchers are developing causal inference methods to quantify the effects of actions within heterogeneous observational data [2–5]. One approach to causal inference leverages “natural experiments,” fortuitous occurrences that serve to segment a population into a treatment group that was affected by a change and a control group that was not. Agrist [6], for example, examined the impact of military service on individual’s lifetime earnings using the Vietnam War draft lottery to separate individuals who performed military service (the treatment group) from those who did not serve (the control group). Comparing these populations allowed Agrist to estimate the effect of military service on earnings. Since this pioneering study others have used abrupt changes—raising the legal drinking age [7], changing the minimum wage [8], or modifying a website’s user interface [9]—to infer the effects of policies [10, 11].

Identifying natural experiments requires creativity and luck, which has made this an underutilized tool in the social sciences. One of the main difficulties is to identify exogenous events that may significantly affect a population. This task, however, can be made easier with change point detection, a method that detects events that suddenly modify a feature distribution. Once these change points are found, researchers can look within a narrow time range for events that contributed to these changes and use regression discontinuity to measure their effects. Change point detection, however, is challenging because social data is typically massive (many people) but sparse (few observations per individual), high dimensional (many features), dynamic, and noisy.

A growing body of research has proposed methods to detect change points, from simple approaches based on cumulative summation [12, 13] to more sophisticated methods based on Markov models [14, 15] and Bayesian statistics [16]. Many of the existing methods, however, are bespoke to problem domains or are only meant for time series. Bayesian approaches, for example, usually need data to follow a particular set of distributions. Moreover, while these methods will identify where the change occurs, many are not able to quantify estimation error or their confidence in the change. Despite the strengths and successes of existing change point detection methods, there is a critical need for an accurate and general purpose method that can be applied to various data, including high-dimensional sparse data like video, audio, and EKG sensor signals.

### Our contribution

We describe *Meta Change Point Detection* (MtChD), a self-supervised method for detecting changes in high dimensional data. The method extends on a confusion-based training meta-model used to detect phase transitions in matter [17] by introducing a mathematical model of classification accuracy to more precisely infer both when the change occurs and the fraction of data affected by change [18]. The method labels data as occurring before (0) or after (1) each candidate change point and trains a classifier to predict the labels. A mathematical model is then trained to estimate classification accuracy as a function of a feature, *t*. The model parameters provide an estimate of the expected change point as well as the fraction of data affected, which is a proxy of change confidence: we trust the change point more if a large fraction of data is affected [18].

We apply MtChD to a range of data, both synthetic and real-world, to demonstrate that it has low bias under a wide range of conditions and accurately detects changes in noisy and high-dimensional data, including images and text. Our method uses standard classifiers, such as a random forest or a multilayer perceptron (MLP), to outperform state-of-the-art change detection methods, even on sparse, noisy, and incomplete real-world data. We show that our method accurately infers events in real-world data that are useful for discovering regression discontinuities that represent potential natural experiments. We show examples our method uncovers, including the impact of COVID-19 lockdowns on air pollution and website policy changes on student performance in a learning platform. Due to MtChD’s flexibility, accuracy and robustness, the proposed framework significantly advances the state-of-the-art in change point detection, thereby opening new opportunities for data-driven discovery.

The rest of the paper is organized as follows. First, we review research on change point detection. Next, we present details of our confusion-based training method and derive the mathematical model of accuracy. We thoroughly evaluate the performance and robustness on an array of synthetic and real-world datasets, and then apply RDD on the discovered real-world events.

## Related work

### Change point detection

Change point detection has a long history. An early method, called CUSUM [12], can detect changes in univariate time serie data but assumes the data follows a normal distribution with known parameters and the method only detects changes in the mean. A major improvement over CUSUM are the general likelihood ratio (GLR) test-based algorithms [19–22]. The GLR-based algorithms seek to reject a null hypothesis that observations before and after a proposed change point follow the same parameterized distribution. Wherever this null hypothesis is least likely compared to a two-distribution hypothesis is the estimated change point. With the help of advanced search algorithms [23–27], new change point detection algorithms based on cost functions can detect multiple (rather than single) change points. A collection of cost functions and search algorithms is available as a Python library called *ruptures* [23].

Alternate methods for change point detection include hidden Markov model (HMM) and alternative code function approaches. Change point detection can, for example, be formulated as a state transition in a HMM [15]. There are also Bayesian change point detection methods [16, 28–30]. Moreover, apart from cost function-based change point detection, there exists penalized quasi-likelihood [31] and kernel methods [32]. Unsupervised Change Analysis is a method most closely aligned with ours [33] as it uses a similar labeling method. But the paper focuses on explaining changes and not quantifying the change point.

Existing methods have significant drawbacks. First, methods are not generalizable. For example, kernel-based support vector machine methods do not perform as well as deep learning methods on image datasets [34]. Moreover, the computational complexity of segmentation-based methods and Bayesian methods scale quadratically with data length, which makes these methods ineffective for long datasets. Although some methods, such as PELT segmentation [27], scale linearly, certain assumptions must be made about the data and cost function.

Our method improves on previous methods in several ways. First, it can estimate the fraction of data affected by change, a proxy of change confidence. Moreover, our method can handle many data forms and be applied to many supervised learning models. Finally, our method scales almost linearly with respect to the length of data. This is because our method requires a small number of training rounds (usually no more than 20) for the candidate change points.

### Natural experiments

Natural experiments have become a popular tool to measure the effects of treatments and policy changes. Agrist’s pioneering study [6] used Vietnam War draft lottery as a natural experiment to measure the effect of military service on individual’s lifetime earnings. The lottery created a quasi-random assignment, putting some individuals in the treatment group (drafted) and others in the control (not drafted). Other studies have since leveraged abrupt exogenous changes unrelated to an outcome to separate the population into treated (after the change) and untreated (before the change) groups and compare outcomes for these groups. Regression discontinuity design (RDD), a framework for measuring effects of changes, is a subcategory of natural experiments [35]. Studies used natural experiments to explore the effect of raising the minimum drinking age on traffic accidents [7], the effect of minimum wage on employment [8], and impact of the prenatal environment on individual’s future health [36]. However, identifying natural experiments requires creativity and insight on the part of researchers to connect some random event in the natural world to their research question. Our method offers a systematic approach to sift through observational data to identify candidates for causal inference, such as RDDs.

## Methodology

### Problem statement

Assume we have data of the form $(X_{i}, t_{i}), i = 1,\dots ,n$, where *X* is an arbitrarily high dimensional vector and *t* is a different data dimension, such as time. We refer *t* as the *indicator* and look for a change point in *t*. Assume there is a change at $t_{0}$ such that some data before the change and some after the change have different distributions. In many datasets, however, only a fraction of data, $0\le \alpha \le 1$, may show observable changes. *Our goal is to infer the change point,* $t_{0}$*, and the fraction of data that undergoes the change,* *α, given the observations* $(X_{i}, t_{i})$*.*

### Step 1: Confusion-based training

Similar to [17], we assume a candidate change point $t=t_{a}$ and label the observed data before $t_{a}$ as belonging to class $\tilde{y}_{i} = 0$, and the data after $t_{a}$ as class $\tilde{y}_{i} = 1$.

We then train a classifier to predict the labels $\tilde{y}_{i}$ from the features $X_{i}$. We plot the accuracy of the classifier as a function of $t_{a}$ for the entire range of indicator *t*. In case a true change point exists in the observed range of *t*, the accuracy vs. $t_{a}$ curve will significantly increase over the baseline accuracy, which is the majority class ratio of labels *ỹ*. The shape of the curve will be affected both by the actual change point, $t_{0}$, and the fraction of data points affected by change, *α*. Any classifier can be used — we use random forest and MLPs in applications described in this paper. For each candidate change point $t_{a}$, classifiers are trained on random splits of 50% of data, validated on 30%, and tested on 20%. The test set is used to judge the accuracy of the learned models for each $t_{a}$. This step is known as confusion-based training.

Accuracy varies significantly with $t_{a}$: near the beginning and end of the dataset, accuracy is nearly 1 (we get high accuracy since a large portion of data is labeled “0” or “1”), but accuracy drops when we move away from these extremes. If $t_{a}$ is near $t_{0}$, the accuracy will again be high because in this case, the created labels *ỹ* matches the true change in data. Thus an accuracy versus $t_{a}$ plot will have a “W” shape [17].

### Step 2: Modeling accuracy vs. $t_{a}$ curve

We show that by modeling this accuracy curve we can better infer $t_{0}$ and, in contrast to Step 1 alone, we can also estimate *α*. We assume that the change happens instantaneously to simplify calculations. We model the CDF of *t*, $F(t)$, using a cubic spline of the emperical CDF, $\tilde{F}(t)=1/T\sum_{i}1(t_{i}\leq t)$. (Other options should not significantly affect the results.) Data *X* can fall into three categories (or three distinguishable distributions): (a) a distribution that does not change, $S_{u}$, which comprises $1-\alpha $ of all data; (b) a distribution before the change ($t\leq t_{0}$), $S_{0}$; (c) a distribution after the change ($t>t_{0}$), $S_{1}$. We do not know these distributions *a priori* but we assume the trained classifier will be able to distinguish these distributions using data *X*.

Assume that the distribution of *t* is independent of the event $X \in S_{u}$, $X \in S_{0}$ or $X \in S_{1}$. With real change point locate at $t_{0}$, given any *t*, we assume that among *α* fraction of data affected by change, $\theta (t-t_{0})$ fraction of data belongs to $S_{1}$ and $1-\theta (t-t_{0})$ fraction of data belongs to $S_{0}$. Here $\theta (\cdot )$ is the Heaviside step function, repesenting an instantaneous change, but a gradual change can be modeled using a sigmoid-like function. We can estimate the fractions of data in $S_{u}$, $S_{0}$, and $S_{1}$ as

1 $$\begin{aligned} &P_{S_{u}} = 1 - \alpha, \end{aligned}$$

2 $$\begin{aligned} &P_{S_{0}} = \alpha F(t_{0}), \end{aligned}$$

3 $$\begin{aligned} &P_{S_{1}} = \alpha \bigl(1-F(t_{0})\bigr). \end{aligned}$$

Recall we label data as “0” if $t_{a} \leq t$ and “1” otherwise. Given candidate change point $t_{a}$, $P_{S_{u,0}}=(1-\alpha )F(t_{a})$ of data in $S_{u}$ is labeled “0” and $P_{S_{u,1}}=(1-\alpha )-P_{S_{u,0}}$ is labeled “1”. On top of this, for a data point in $S_{u}$, the expected predicting accuracy should be $\frac{1}{1-\alpha}\max (P_{S_{u,0}}, P_{S_{u,1}})$. Similarly, we can calculate the ratio of data labeled as “0” or “1” in $S_{0}$ and $S_{1}$, respectively. We can calculate for $S_{1}$, which has fraction $P_{S_{1}} = \alpha (1-F(t_{0}))$, the fraction of data labeled “1” as

4 $$ P_{S_{1,1}}= \max \bigl\{ \alpha \bigl[F(t_{a})-F(t_{0}) \bigr], 0\bigr\} . $$

And the fraction of data labeled “0” is

5 $$ P_{S_{1,0}} = P_{S_{1}} - P_{S_{1,1}} = \alpha \bigl(1-F(t_{0})\bigr) - P_{S_{1,1}}. $$

The expected predicting accuracy for $S_{1}$ is thus $\frac{1}{\alpha (1-F(t_{0}))}\max (P_{S_{1,0}}, P_{S_{1,1}})$. Finally, $S_{0}$ has a fraction of $P_{S_{0}} = \alpha F(t_{0})$. The total fractions of data labeled “0” in both $S_{0}$ and $S_{1}$ is

6 $$ P_{S_{1,0}} + P_{S_{0,0}}=\alpha F(t_{a}). $$

This gives $P_{S_{0,0}} = \alpha F(t_{a}) - P_{S_{1,0}}$. Therefore the fraction in $S_{0}$ incorrectly labeled as “1” is

7 $$ P_{S_{0,1}} = P_{S_{0}} - P_{S_{0,0}} = \alpha F(t_{0}) - P_{S_{0,0}}. $$

The expected predicting accuracy for data point in $S_{0}$ is then $\frac{1}{\alpha F(t_{0})}\max{( P_{S_{0,0}}, P_{S_{0,1}})}$.

We then utilize the results above to estimate the accuracy as a function of $t_{a}$ using the average predicting accuracy in $S_{u}$, $S_{0}$ and $S_{1}$ weighted by the fraction of these three sets. Namely,

8 $$ \tilde{A}cc(t_{a}) = \max{( P_{S_{u,0}}, P_{S_{u,1}} )} + \max{( P_{S_{0,0}}, P_{S_{0,1}} )} + \max{( P_{S_{1,0}}, P_{S_{1,1}} )}. $$

These variables only depend on empirically estimated CDF, $F(t)$, and the free parameters $t_{0}$ and *α*. We therefore do not need to know the distributions of $S_{0}$, $S_{1}$ and $S_{u}$. To estimate $t_{0}$ and *α*, we can do a grid search and use a mean squared error cost function to fit the observed accuracy. The standard error of *α* and $t_{0}$ are estimated via multiple random splits of data. The source code to is available on our GitHub repository.^1^

### State-of-the-art

We compare our method against state-of-the-art change detection methods. These methods can be divided into two groups, optimal segmentation algorithms and Bayesian change point detection. Optimal segmentation algorithms we compare against include dynamic programming (DP) [24], binary segmentation [25], bottom up methods [26], and window based methods [23] with $L_{1}$, $L_{2}$, normal distribution loss and RBF kernel loss functions. These algorithms are implemented in the Python package *ruptures* [23]. We also compare against GLR, which is equivalent to optimal segmentation with a normal distribution likelihood cost function. Bayesian change point detection requires a prior and likelihood function. We used uniform and geometric distributions as priors and applied Gaussian, individual feature model [30], and full covariance model [30] as likelihood functions. We used a Python implementation for Bayesian change point detection available from GitHub.^2^

## Results

We demonstrate the accuracy and robustness of our method on data from a variety of domains. We first apply it to synthetic data to evaluate method’s performance and robustness with respect to noise, then apply it to real-world data to discover changes corresponding to external events. Finally, we illustrate how leveraging regression discontinuities around the newly-discovered changes enables us to estimate effects of events and policies.

### Discovering changes in synthetic data

#### Synthetic “chessboard pattern”

**Figure 1:**
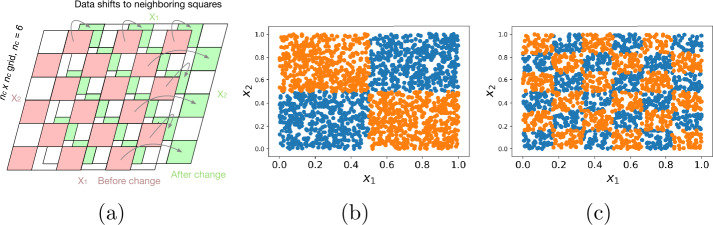
Illustrations of synthetic data (**a**), where observations have two features $x_{1}$ and $x_{2}$. In (**b**) and (**c**), blue dots represent data points which satisfy $t\leq t_{0}$ and orange dots are for $t> t_{0}$. (**b**) and (**c**) are for $n_{c}=2$ and $n_{c}=6$, respectively. For fixed data size *N*, as $n_{c}$ increases, the number of data points in each square decreases

In this experiment, we generate two-dimensional numeric data in a chessboard pattern, with two features $x_{1}$ and $x_{2}$, each in the range $[0, 1]$, as shown in Fig. 1. At a time $t_{0}$, data points spread uniformly at random within the blue squares of a $n_{c}\times n_{c}$ chessboard move to the orange squares of the chessboard. Mathematically, for $n_{c}\times n_{c}$ chessboard, the data generated satisfies the following condition,

9 $$\left(\lfloor n_{c}\cdot x_{1}\rfloor +\lfloor n_{c}\cdot x_{2}\rfloor \right)\mathrm{mod}2=1(t>t_{0}).$$

For first part of this experiment, we set $t_{0} = 0.5$ and the size of the data $N = 8K$. We use different arrangements of the chessboard, $n_{c} = \{2, 4, 6, 8, 10\}$. For higher $n_{c}$, the data is grouped into smaller chess squares with fewer data points per square. For second part of this experiment, we fix $n_{c} = 6$ (a six by six chessboard) and we vary $t_{0}$ between 0.2 and 0.8.

We repeat our method and comparing algorithms for 6 times on random data splits. For the optimal segmentation methods, we randomly sample 70% of data in each trial. Due to computational limitations, we only sample 18.8% of data (around 1.5K) for Bayesian change point detection.

The results are shown in Table 1. In the tables and figures, *μ* and *σ* are the estimated mean and standard error of parameters, respectively. For our method, *α* represents the fraction of data changed. We see that for small $n_{c}$, optimal segmentation methods perform as well as ours, but for $n_{c} \ge 6$, our method outperforms comparing methods. Of the two classifiers used by our method, random forest performs better.

**Table 1:** A comprehensive comparison of the performance of the proposed method against two types of state-of-the-art methods: optimal segmentation and Bayesian change point detection on synthetic data. *MtChD(RF)* is our method with a random forest classifier; *MtChD(MLP)* is our method with a MLP classifier. *DP* + *Normal (GLR eq.)* is DP segmentation method used with normal loss function, which is equivalent to GLR test that assumes a multivariate normal distribution. Six combinations of optimal segmentation methods are listed. *DP* is dynamic programming segmentation algorithm, *BinSeg* is binary segmentation, *Window* is window-based change point detection, and *BottomUp* is Bottom-up segmentation. The cost functions used are *RBF* (RBF kernel), *L1* ($L_{1}$ loss function), and *L2* ($L_{2}$ loss function). The last four rows are for Bayesian change point detection with a *uniform* prior or *Geo* (geometric) prior. *Gassusian* stands for Gaussian likelihood function, *IFM* is the individual feature model [30], and *FullCov* is the full covariance model [30]. $\mu (t_{0})$ and $\sigma (t_{0})$ are the mean value and standard deviation of inferred change point and $\mu (\alpha )$ and $\sigma (\alpha )$ are the mean value and standard deviation of inferred *α*. Bold values indicate change points that are closest to the correct value

	$n_{c}$	2	4	6	8	10	6	6	6	6
	$t_{0}$	0.5	0.5	0.5	0.5	0.5	0.2	0.4	0.6	0.8
MtChD (RF)	$\mu (t_{0})$	**0.5002**	**0.4983**	**0.4976**	**0.5000**	**0.4959**	**0.1950**	**0.3937**	**0.6014**	**0.8020**
	$\sigma (t_{0})$	**0.0025**	**0.0017**	**0.0033**	**0.0005**	**0.0049**	**0.0047**	**0.0052**	**0.0023**	**0.0022**
	*μ*(*α*)	**0.9494**	**0.9137**	**0.8562**	**0.7604**	**0.6573**	**0.6503**	**0.8429**	**0.8316**	**0.6580**
	*σ*(*α*)	**0.0077**	**0.0041**	**0.0119**	**0.0220**	**0.0156**	**0.0346**	**0.0076**	**0.0133**	**0.0276**
MtChD (MLP)	$\mu (t_{0})$	**0.5027**	**0.5003**	0.5262	0.5084	0.5772	0.5649	0.4095	0.5962	0.5372
	$\sigma (t_{0})$	**0.0027**	**0.0039**	0.0173	0.0962	0.0569	0.0450	0.0258	0.0668	0.1315
	*μ*(*α*)	**0.9589**	**0.8289**	0.6249	0.0048	0.0086	0.0045	0.4906	0.3950	0.0171
	*σ*(*α*)	**0.0095**	**0.0366**	0.0710	0.0068	0.0080	0.0035	0.0534	0.1112	0.0202
Naive Confusion (RF)	$\mu (t_{0})$	0.4965	0.5017	0.4974	0.4975	0.4973	0.2271	0.4255	0.5235	0.5436
	$\sigma (t_{0})$	0.0018	0.0019	0.0004	0.0001	0.0001	0.0382	0.0312	0.0229	0.0900
DP + Normal	$\mu (t_{0})$	**0.5003**	**0.5006**	0.5212	0.7238	0.5971	0.2441	0.4578	0.5885	0.8108
(GLR eq.)	$\sigma (t_{0})$	**0.0004**	**0.0005**	0.0204	0.2762	0.3374	0.0377	0.0447	0.0266	0.0288
DP + RBF	$\mu (t_{0})$	**0.5002**	**0.5001**	0.5673	0.9495	0.3071	0.3740	0.4234	0.5827	0.8355
	$\sigma (t_{0})$	**0.0004**	**0.0019**	0.0684	0.0679	0.2392	0.2840	0.1893	0.0246	0.0654
DP + L2	$\mu (t_{0})$	0.9510	0.9875	0.3515	0.8584	0.5143	0.4451	0.3183	0.3104	0.2917
	$\sigma (t_{0})$	0.0099	0.0062	0.2399	0.2734	0.4006	0.3481	0.4417	0.4252	0.3778
DP + L1	$\mu (t_{0})$	0.9569	0.5313	0.5809	0.6053	0.4015	0.5526	0.1277	0.4916	0.2114
	$\sigma (t_{0})$	0.0070	0.2660	0.1677	0.4027	0.3308	0.4467	0.1873	0.3832	0.3312
BinSeg + RBF	$\mu (t_{0})$	**0.5002**	**0.4995**	0.5701	0.7663	0.5635	0.3133	0.3850	0.6049	0.7258
	$\sigma (t_{0})$	**0.0002**	**0.0011**	0.0502	0.3205	0.2190	0.3285	0.3702	0.1506	0.2715
Window + RBF	$\mu (t_{0})$	0.4391	0.5653	0.2960	0.5699	0.2444	0.4746	0.5654	0.7964	0.3987
	$\sigma (t_{0})$	0.1364	0.2210	0.2139	0.1738	0.1012	0.2436	0.2459	0.2223	0.3159
BottomUp + RBF	$\mu (t_{0})$	**0.5002**	0.4581	0.4500	0.6821	0.4947	0.4271	0.5213	0.4602	0.5861
	$\sigma (t_{0})$	**0.0008**	0.1477	0.3655	0.2879	0.3144	0.3059	0.2149	0.2885	0.2953
Uniform + Gaussian	$\mu (t_{0})$	0.5474	0.5429	0.3915	0.4717	0.5429	0.6171	0.7546	0.5210	0.5196
	$\sigma (t_{0})$	0.2299	0.3010	0.1567	0.2265	0.2159	0.2842	0.2203	0.1549	0.3386
Uniform + IFM	$\mu (t_{0})$	0.9969	0.9942	0.9973	0.9975	0.9975	0.9986	0.9958	0.9973	0.9985
	$\sigma (t_{0})$	0.0031	0.0030	0.0020	0.0015	0.0030	0.0015	0.0049	0.0026	0.0012
Uniform + FullCov	$\mu (t_{0})$	0.4985	0.5089	0.9986	0.9976	0.9989	0.9930	0.9280	0.9982	0.9974
	$\sigma (t_{0})$	0.0002	0.0163	0.0006	0.0010	0.0009	0.0098	0.1593	0.0020	0.0038
Geo + Gaussian	$\mu (t_{0})$	0.0282	0.0271	0.0286	0.0323	0.0278	0.0326	0.0340	0.0312	0.0254
	$\sigma (t_{0})$	0.0044	0.0018	0.0044	0.0054	0.0037	0.0063	0.0034	0.0051	0.0037

#### Synthetic images

**Figure 2:**
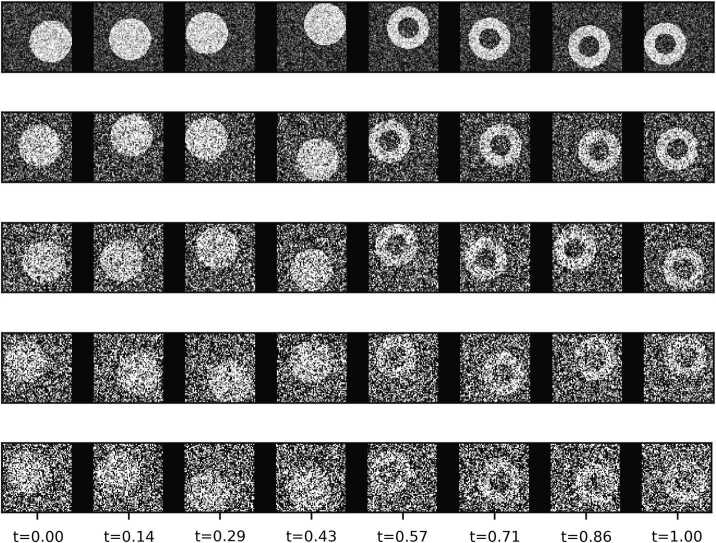
Example time series of synthetic images that change at $t_{0}=0.5$ when a solid circle changes to a hollow circle. From top to bottom, each row shows images with an increasing noise level $\sigma = 0.2, 0.4, 0.6, 0.8$ and 1.0

Our method can also identify changes in diverse high-dimensional data, such as text [18] and images. To illustrate this we generate a series of synthetic 64 by 64 pixel gray scale images that qualitatively change at $t_{0}=0.5$ from solid to hollow circles (Fig. 2). These images can represent, for example, organisms that were originally alive and then died; thus our task would be to determine the moment an organism died, a finding that is very useful in the field of survival analysis [37]. The gray scale of the solid and hollow circles is $\gamma = 0.8$ and the gray scale of the background is $\gamma = 0.2$. To create more realistic data, we position the circles randomly within the image and inject different levels of Gaussian noise to model poor quality data. After adding noise, pixel grey scale values are truncated to the range $[0.0, 1.0]$. We also assign each image a random time *t* uniformly distributed between 0 and 1. For every noise level, we generated a dataset with 4,000 images respectively.

We check the robustness of the estimated change point against noise. Table 2 shows the inferred change point and estimated value of *α* as a function of noise for the synthetic image data. Due to spatial correlation of image data and the superior predicting power of CNN classifier, the change point inferred is close (often not statistically significantly different) to the true change point and *α* is close to 1.0, even for very noisy image frames. Alternative methods were infeasible because of the high-dimension and large data size.

**Table 2:** Change points inferred for noisy synthetic images. The true value of change point is $t_{0} = 0.50$ where solid circles change into hollow circles with different levels of noise

Noise	0.20	0.40	0.60	0.80	1.00
$\mu (t_{0})$	0.5048	0.5087	0.5253	0.5155	0.5380
$\mu (t_{0})-t_{0}$	0.0047	0.0086	0.0237	0.0191	0.0398
$\sigma (t_{0})$	0.0028	0.0043	0.0027	0.0111	0.0246
*μ*(*α*)	0.9612	0.9787	0.9298	0.9609	0.8781
*σ*(*α*)	0.0278	0.0139	0.0083	0.0361	0.0717

### Discovering changes in real-world data

We now demonstrate the ability of MtChD to identify changes in real-world data.

#### Covid-19 air quality

We first apply our method to air pollution data to see if pollution drops around the time the COVID-19 pandemic occurred. We collected air quality data daily from January 1 to May 26, 2020 for major U.S. cities from AQICN (aqicn.org). This data includes daily concentrations of nitrogen dioxide, carbon monoxide, and fine particulates less than 2.5 microns across (PM2.5), totalling 4.3K observations for 37 cities across the U.S. once missing data are removed. We also include population within 50 km of the city as a feature because people within this area may have contributed to the concentration of pollutants. We can use our model to determine when the change started, and compare these results to the gold standard: the date stay-at-home orders were issued by states. These orders limited business and commercial activity, which likely lead to the dramatic decline in pollution, and therefore act as the ground truth external events for RDDs. The earliest such order was announced in California on March 19, 2020 and the latest in South Carolina on April 7.

**Figure 3:**
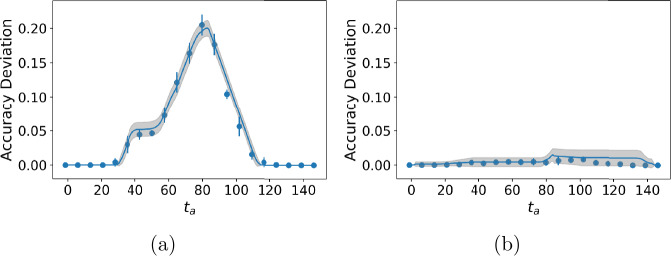
Accuracy deviation curve for *COVID-19 Air* data. (**a**) Using random forest classifier; (**b**) Using a MLP classifier. The scatter points are accuracy deviation measured on testing set and the solid lines are fitted using the proposed accuracy deviation model

We compare our method to state-of-the-art algorithms in Table 3. Our method is the only one that inferred a reasonable change point for the data of March 21, 2020 ± 3 days, roughly in the middle of all state stay-at-home orders. We show accuracy deviation for MtChD in Fig. 3. A random forest classifier gives better accuracy than MLP and the mathematical model fits accuracy deviation well. Although our method can work with any classifier, the performance on a given dataset can be improved by choosing a classifier that best fits the data. Some empirical ways to determine which classifier to use is (a) choosing the classifier that gives the largest accuracy deviation or (b) choosing the classifier that gives the highest *α*.

**Table 3:** A comprehensive comparison of our method with previous methods on real world datasets, *COVID-19 Air* and *Khan Academy*. We use the same abbreviations as in Table 1. For *COVID-19*, the measure of $t_{0}$ is number of days since 01/01/2020. For *Khan Academy*, the measure of $t_{0}$ is Unix timestamp, namely, number of seconds since midnight 01/01/1970. Correct values are roughly 80 days for COVID-19 air data, and $1.365 \times 10^{9}$ seconds for Khan Academy data. Bold values indicate change points that are closest to the correct value

		COVID Air	Khan
		Time (day)	Time (sec)
MtChD(RF)	$\mu (t_{0})$	**80.0829**	$\mathbf{1.3703e}{\boldsymbol{+}}\mathbf{09}$
	$\sigma (t_{0})$	**2.9713**	$\mathbf{2.6992e}{\boldsymbol{+}}\mathbf{05}$
	*μ*(*α*)	**0.4164**	**0.2803**
	*σ*(*α*)	**0.0392**	**0.0029**
MtChD(MLP)	$\mu (t_{0})$	99.5820	$\mathbf{1.3694e}{\boldsymbol{+}}\mathbf{09}$
	$\sigma (t_{0})$	99.5820	$\mathbf{1.3694e}{\boldsymbol{+}}\mathbf{09}$
	*μ*(*α*)	0.4843	**0.1491**
	*σ*(*α*)	0.3264	**0.0173**
DP + Normal	$\mu (t_{0})$	71.8333	1.3577e+09
(Normal GLR eq.)	$\sigma (t_{0})$	0.3727	2.2059e+07
DP + RBF	$\mu (t_{0})$	37.1667	1.3763e+09
	$\sigma (t_{0})$	25.5761	9.4481e+06
DP + L2	$\mu (t_{0})$	70.1667	1.3679e+09
	$\sigma (t_{0})$	53.8911	1.0014e+07
BinSeg + RBF	$\mu (t_{0})$	1.0000	1.3741e+09
	$\sigma (t_{0})$	0.0000	8.9074e+06
Window + RBF	$\mu (t_{0})$	55.0000	1.3587e+09
	$\sigma (t_{0})$	0.0000	1.2031e+07
BottomUp + RBF	$\mu (t_{0})$	54.0000	1.3528e+09
	$\sigma (t_{0})$	0.8165	1.2960e+06
Uniform + Gaussian	$\mu (t_{0})$	96.9167	1.3439e+09
	$\sigma (t_{0})$	37.5859	4.2047e+06
Uniform + IFM	$\mu (t_{0})$	−0.5833	1.3564e+09
	$\sigma (t_{0})$	0.8858	1.5300e+07
Uniform + FullCov	$\mu (t_{0})$	0.0000	1.3591e+09
	$\sigma (t_{0})$	0.6455	1.6176e+07
Geo + Gaussian	$\mu (t_{0})$	8.1667	1.3396e+09
	$\sigma (t_{0})$	8.9334	2.9504e+05

#### Khan academy

As a second example, we apply our method to the learning platform Khan Academy (khanacademy.org), which offers courses on a variety of subjects where students watch videos and test their knowledge by answering questions. The Khan Academy platform had undergone substantial changes to its user interface around April 1, 2013 (or $1.3648\times 10^{9}$ in Unix epoch time) [38], which affected user performance. This change acts as a ground truth event we want to detect. After discovering this event, we can take regressions of scores before and after the event and determine if this policy significantly changes student performance scores via a RDD.

Data was collected by Khan Academy over the period from June 2012 to February 2014 and contains 16K questions answered by 13K students totalling 681K data points. Despite the large number of students, the data is very sparse: the vast majority of students were typically active for less than 20 minutes and never returned to the site. The performance data records whether the student solved the problem correctly on their *first attempt* and without a hint. When the user failed, they were able to attempt the problem again, and the *number of attempts* made on a problem is recorded. Additional features recorded include the *time since the previous problem*, the *number of problems* in a student session, and the *number of sessions*. Segmentation methods implemented in *ruptures* are not memory efficient, therefore we only sample 0.5% of the data (about 3.5K entries) uniformly at random. For Bayesian change point detection, we sampled around 1.6K data points uniformly at random.

**Figure 4:**
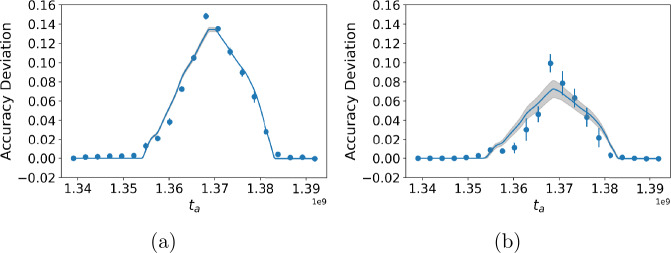
Accuracy deviation curve for *Khan Academy* data. (**a**) Using random forest classifier; (**b**) Using a MLP classifier

Both our method and optimal segmentation algorithms can identify the change from user performance data (Table 3), although optimal segmentation algorithms have larger error. Bayesian change point detection does not give a reasonable change point for this data. The accuracy deviation curve is shown in Fig. 4. The random forest classifier and MLP classifier have comparable performance when used to estimate change points.

### Measuring effects of changes via regression discontinuity design

We demonstrate how we can use regression discontinuity design to measure the effects of changes on the population. Automatically discovered changes can therefore help uncover potential natural experiments in data.

#### Persistence and performance in learning on Khan academy

Our analysis uncovered an abrupt change around April 2013 in the Khan Academy data (Sect. 4.2.2). The change only affected user performance in a fraction of all sessions, quantified by parameter *α* in Table 3. This change was likely due to a major redesign of the platform’s user interface [38], although we do not know exactly what changed. We found no indication that the population was any different before and after the change. Therefore, the April 2013 change could be used for a RDD, with some users “assigned” quasi-randomly to visit the platform before the interface change and some after. This created an effective control condition (before the change) and treatment condition (after the change). The external event allows us to control for some of the confounders when investigating correlates of performance in learning platforms. Specifically, comparing treated group to the controled helps identify the link between persistence (working longer on problems first answered incorrectly) and performance (answering the problem correctly on the first attempt).

**Figure 5:**
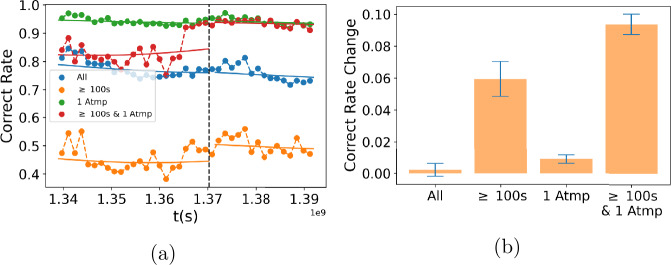
Performance in Khan Academy. (**a**) Performance vs. time in Khan Academy data for problems binned with duration and number of attempts. (**b**) Change of performance for binned data

**Figure 6:**
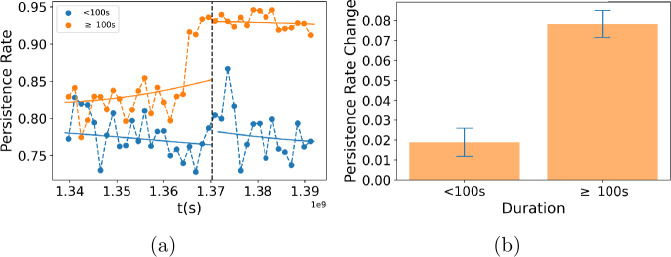
Persistence in Khan Academy. (**a**) Persistence rate vs. time in Khan Academy data for long (≥ 100 sec) and short (< 100 sec) duration questions. (**b**) Change of persistence rate for long and short duration problems

Figure 5(a) shows average performance over time, measured as the fraction of problems the user solved correctly on their first attempt. Performance decreases gradually for all users over the two-year period (blue line), despite seasonal variation. However, for users working on problems that take more than 100 seconds to answer, i.e., hard problems, performance increases after the change (orange line). To estimate the effect of the change, we binned the data and fit the outcomes before and after the change as functions of time using two kernel models (see Appendix A.2 for details). The effect is strongest in users who solve hard problems correctly on their first attempt (Fig. 5(b)). At the same time, users became more *persistent*, i.e., more likely to continue working on a problem they did not solve correctly on the first attempt (Fig. 6(a)). The effect is bigger for users working on hard problems (Fig. 6(b)). Thus, the change had two effects: it made users working on hard (to them) problems more persistent, and this improved their performance on other hard problems, i.e., made them more likely to correctly solve these problems on the first try. Improvement in performance for these users was large, ∼10%, which corresponds to *a full letter grade* in a class setting. Psychological studies have identified traits, such as conscientiousness or grit, that allow some people to practice a skill so as to achieve mastery [39]. Our study supports the link between persistence and improved performance.

#### Covid-19 lockdowns reduced air pollution

**Figure 7:**
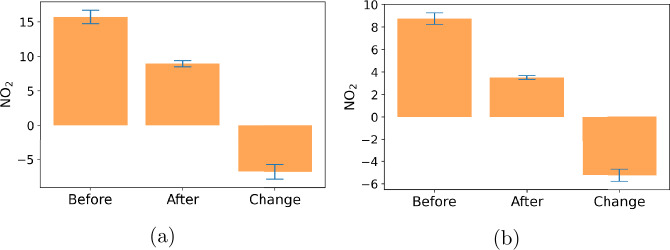
Averaged and change of nitrogen dioxide levels before and after Mar. 21, 2020. (**a**) For Manhattan, (**b**) For San Francisco

We detect a change on Mar. 21, 2020 in the COVID-19 Air Quality data (Sect. 4.2.1). The change is consistent with the dates of the COVID-19 lockdown orders in the US, in which people had to stay at home to reduce the spread of the disease. We calculated the change in nitrogen dioxide levels before and after Mar. 21, 2020 as shown in Fig. 7. For both Manhattan and San Francisco, nitrogen dioxide levels drop significantly (by around 5 ppb) after the lockdown. The reduction in air pollution is due to reduced traffic after the lockdown. Our findings of the date and effect of the change are confirmed by Venter et al. [40].

## Discussion

We introduce *Meta Change Point Detection* (MtChD), a novel method to detect changes in high dimensional data. The method identifies changes in a wide range of data, from tabular to images. Moreover, it gives us the fraction of data changed, which we find can act as a confidence metric. Our comprehensive experiments validated the method on synthetic and real-world data that are difficult for other methods, and showed that it can robustly identify changes in sparse and noisy data. We also demonstrate that our method has low bias with higher accuracy than competing state-of-the-art methods, and efficiently handles large datasets.

MtChD can be used in tandem with regression discontinuity designs to discover effects of policies within observational data. By accurately estimating when a change occurs, we can uncover plausible exogenous events that produce these changes, and then use RDDs to determine average treatment effects of the event, thereby discovering natural experiments in data. Importantly, RDDs assume unconfoundedness: the treatment (i.e., change) is unaffected by the outcome variable. Therefore, RDDs on the change points themselves would not be methodologically sound. Instead, the method offers candidate events and additional research would then reveal what is an appropriate exogenous event and what features are confounded by this change. Therefore, our method substantially reduces research time needed to detect natural experiments.

We illustrate this idea by discovering important events in empirical data. Namely, by applying the change point detection, we identify a change in user performance on Khan Academy. We discover that for long problems, users are both more likely to be persistant and perform *a full letter grade* better. This finding is consistent with the notion that persistent people perform better [39]. It appears that simply by encouraging users to keep working on problems they find challenging (i.e., they failed to solve them on the first attempt), could make these users more successful later on. Our findings therefore hint that user interface design choices might make people more persistent.

Our method helps researchers automatically detect natural experiments otherwise hidden in high-dimensional empirical data [41]. Determining which dimensions produce causal effects is an ongoing problem, especially when the change may be heterogeneous across conditions, as in the case of Khan Academy [42].

## Data Availability

All code and synthetic data (including code to generate synthetic data) is available at https://github.com/yuziheusc/confusion_multi_change. COVID-19 Air dataset is avialable to at https://aqicn.org/data-platform/covid19/. US census dataset is avialable at https://data.census.gov/cedsci/. The processed and cleaned version of the data is available from the corresponding author upon request. Khan Academy dataset is available from Khan Academy but restrictions apply to the availability of these data, which were used under license for the current study, and so are not publicly available. Data are however available from the authors upon reasonable request and with permission of Khan Academy.

## Appendix

### 1.1 Details of confusion based training

We use four hidden layers for the MLP, each with 64 neurons. We chose the ReLU activation function and the maximum number of epochs for training is 100. The random forest classifier uses 100 decision trees with a maximum depth of 32. Entropy is used as the splitting criterion. To detect changes in video data, a convolutional neural network (CNN) is used with six convolutional layers. The dimensions of each layer are 3 by 3, and the number of filters in each layer are 32, 32, 64, 64, 128, and 128. After the second, fourth and the sixth convolutional layer, max pooling and drop out is performed. The kernel size for max pooling is two and stride two, while the drop out ratio is 0.20. The output of the convolutional layers are sent into a fully connected neural network with one hidden layer and 64 neurons. A ReLU activation function was also used for this neural network and the model was trained for 30 epochs.

### 1.2 Kernel regression for effect estimation

We used kernel regressions with RBF kernels to model the average outcomes (*persistence rate* and *correct rate*) as functions of time. Namely,

10 $$ y(t) = \sum_{j} k(t, t_{j})\cdot y_{j} = \sum_{j} \exp \bigl(-\gamma (t-t_{j})^{2}\bigr) \cdot y_{j}. $$

Variable *t* is first standardized. We use $\gamma = 1$. To accelerate the calculation, we set cutoff for kernel weights $k(t, t_{j})$ equals 0.05. For binned data in Fig. 6 and Fig. 5, we perform a kernel regression for each bin, respectively.

## Footnotes

1 https://github.com/yuziheusc/confusion_multi_change

2 https://github.com/hildensia/bayesian_changepoint_detection

## Abbreviations

RDD: Regression Discontinuity Design
MtChD: Meta Change Point Detection
COVID-19: Coronavirus Disease 2019
CUSUM: Cumulative Sum
GLR: General Likelihood Ratio
HMM: Hidden Markov Model
CNN: Convolutional Neural Network
DP: Dynamic Programming
BinSeg: Binary Segmentation
RBF: Radial Basis Function
RF: Random Forest
MLP: Multi-Layer Perceptron
PM2.5: Fine inhalable particles, with diameters that are generally 2.5 micrometers and smaller
